# Standardization of scan protocols for RT CT simulator from different vendors using quantitative image quality technique

**DOI:** 10.1002/acm2.14484

**Published:** 2024-08-13

**Authors:** Hsiang‐Chi Kuo, Usman Mahmood, Assen S. Kirov, Trevin Trotman, Shih‐Chi Lin, James G. Mechalakos, Cesar Della Biancia, Laura I. Cerviño, Seng Boh Lim

**Affiliations:** ^1^ Department of Medical Physics Memorial Sloan Kettering Cancer Center New York USA; ^2^ Department of Radiation Oncology Memorial Sloan Kettering Cancer Center New York USA

**Keywords:** detectability, iterative reconstruction, NPS, TTF

## Abstract

**Objective:**

To investigate the feasibility of standardizing RT simulation CT scanner protocols between vendors using target‐based image quality (IQ) metrics.

**Method and materials:**

A systematic assessment process in phantom was developed to standardize clinical scan protocols for scanners from different vendors following these steps: (a) images were acquired by varying CTDI_vol_ and using an iterative reconstruction (IR) method (IR: iDose and model‐based iterative reconstruction [IMR] of CT_p_‐Philips Big Bore scanner, SAFIRE of CT_s_‐Siemens biograph PETCT scanner), (b) CT exams were classified into body and brain protocols, (c) the rescaled noise power spectrum (NPS) was calculated, (d) quantified the IQ change due to varied CTDI_vol_ and IR, and (e) matched the IR strength level. IQ metrics included noise and texture from NPS, contrast, and contrast‐to‐noise ratio (CNR), low contrast detectability (d′). Area under curve (AUC) of the receiver operation characteristic curve of d′ was calculated and compared.

**Results:**

The level of change in the IQ ratio was significant (>0.6) when using IMR. The IQ ratio change was relatively low to moderate when using either iDose in CTp (0.1–0.5) or SAFIRE in CT_s_ (0.1–0.6). SAFIRE‐2 in CT_s_ showed a closer match to the reference body protocol when compared to iDose‐3 in CT_p_. In the brain protocol, iDose‐3 in CT_p_ could be matched to the low to moderate level of SAFIRE in CT_s_. The AUC of d′ was highest when using IMR in CT_p_ with lower CTDI_vol_, and SAFIRE in CT_s_ performed better than iDose in CT_p_

**Conclusion:**

It is possible to use target‐based IQ metrics to evaluate the performance of the system and operations across various scanners in a phantom. This can serve as an initial reference to convert clinical scanned protocols from one CT simulation scanner to another.

## 1 INTRODUCTION

Standardization of clinical computed tomography (CT) simulation imaging protocols is indispensable in the modern era of precision medicine. Consistent scanning practices in radiation oncology CT simulation optimize treatment planning by reducing variation in image quality (IQ) and minimizing noise in each scan, thereby, enhancing the confidence of dosimetrists in contouring regions of interest for the most personalized therapeutic approach.^1^, ^2^ With technical advancements and increasing automation, CT scanning protocols have become more complex to consider kV, mAs, slice thickness, pitch, iterative reconstruction (IR), automatic tube control (ATC) for KV and mAs modulation, reconstruction kernel, and so forth. Different vendors have varying hardware, software implementations, and terminology, making standardization of the scanning protocols among vendors challenging. With the recent trend of large hospital networks merging or partnering, clinical physicists may face challenge in adapting the IQ and with managing the differences in radiation dose across scanners from various vendors. Standardization of protocols among different vendors to unify IQ for treatment planning has become more essential to provide quality care to patients in radiation therapy (RT). Li et al.^2^ demonstrated the relationship between IQ and treatment planning quality and proposed a strategy using an image quality index (IQI) to measure contouring accuracy of prostate on an anthropomorphic pelvis phantom and to optimize CT simulation protocol. To standardize scanning protocols between scanners, a set of appropriate metrics derived from CT images need to be determined. The IQ of CT is conventionally quantified by noise, uniformity (homogeneity), MTF (spatial resolution), low contrast, and CT number accuracy as recommended by International Electrotechnical Commission (IEC 61223‐3‐5), American College of Radiology (ACR), and the American Association of Physicist in Medicine (AAPM).^3^, ^4^, ^5^, ^6^ These tests are also adopted by most manufactures in their acceptance test and constancy check with specifications varying between manufactures and models.

The objective of these tests is to provide quantitative system performance metrics in a standard CT water phantom. However, compared to conventional filter back projection (FBP) reconstruction, modern CT utilizes advanced non‐linear IR methods, such as statistics‐based IR, model‐based IR, and artificial intelligent (AI) IR. These IR models typically offer different level of strength that can be customized by end‐users. The noise power spectrum (NPS) measures noise properties, including the magnitude of noise and the spatial correlation of noise in the frequency domain (i.e., texture). It is a superior noise descriptor compared to noise magnitude.^7^ Solomon et al.^8^ used the normalized NPS (nNPS) to match the level of reconstruction kernel between two scanners. Afadzi et al.^9^ utilized NPS and noise texture deviation (NTD) to quantify the effect of IR levels on the IQ of ultra‐low dose chest CT. Frank et al.^10^ applied NPS to investigate the preservation of image texture on low dose chest CT image reconstructed with deep learning. Wang et al.^11^ used conventional image metrics, such the normalized mean square error‐NMSE, normalized cross correlation‐NCC, contrast‐to‐noise ratio (CNR), and spatial nonuniformity (SNU), as the loss function in the deep‐learning architecture for IQ improvement on low‐dose CT simulation in RT. Moreover, AI‐based technologies, which were trained with prior clinical data sets, are used to enhance CT imaging, including IQ improvement, organ delineation, disease diagnosis, or prediction. An IQ metric capable of providing task‐based quantitative evaluation is needed for operational performance assessment.

Task‐based measures of IQ were developed and introduced for CT performance assessment, including the object modulation transfer function (MTF) or task transfer function (TTF) and detectability index (d′). TTF and d′ were used to evaluate IQ of the deep learning reconstructed CT images compared to FBP or IR in phantom.^12^, ^13^ Smith et al.^14^ extended the detectability index to apply for estimating d′ in vivo CT images. The AAPM Task Group 233 report^15^ discusses and summarizes IQ metrics to assess the system and operational performance of CT. In this study, to unify IQ of CT images for treatment planning in a large network, we presented a scheme using the IQ metrics recommended by TG 233 to compare the system and operational performance of scanners from two different vendors and to translate clinical protocols from one CT simulator to another by matching a list of IQ metrics.

## 2 MATERIAL AND METHODS

### 2.1 Schemes and materials for standardization

In this study, a five‐step process was proposed to standardize clinical scan protocols for scanners from different vendors: (a) classify protocols into body and brain protocols, (b) obtain the rescaled NPS, (c) scan images by varying CTDI_vol_ and IR, (d) evaluate the IQ change due to varied CTDI_vol_ and IR, and (e) match the IR strength level. The details of each step are shown below.

The clinical protocols implemented and applied in a Philips Big Bore RT CT scanner (Philips Healthcare, Amsterdam, herein referred to as CT_p_) served as a benchmark for evaluating the IQ of a set of scanned images from a Siemens Biograph Vision‐600 PETCT scanner (Siemens Healthcare, Germany, herein referred to as CT_s_) in comparison to a set of scanned images from the CT_p_ scanner. The CT_p_ and CT_s_ scanners are equipped with a carbon fibers flat top tables conventionally used as CT simulators for the purpose of RT planning. The regular reconstruction kernels were utilized for RT's planning purposes rather than diagnostic purposes at both CT_p_ (standard) and CT_s_ (Hr38/Br38) scanners. This study compared the performance of the two scanners at the brain and body protocols at a range of CTDI_vol_ values and at different reconstruction methods: filter‐backprojection (FBP) and IR. The comparison excluded assessments of ATC, as it was outside the scope of the study. IQ metrics recommended in AAPM's Task Group 233 Report were used for evaluation. The scanned parameters for the brain and body protocols are listed in Table 1. The IR strengths evaluated in this study was the hybrid statistical IR, iDose (level 1−6 in body protocol and level 1−5 in brain protocol), the model‐based iterative reconstruction (IMR) (level 1−3) for the CT_p_ scanner, and SAFIRE (Sinogram Affirmed Iterative Reconstruction, level 1−5), and for the CT_s_ scanner. A 20 cm water phantom and a 30 cm water phantom were used to normalize NPS of brain and body scanned images at two scanners. A Catphan phantom (Catphan 504, The Phantom Laboratory, Salem, NY) was used to scan images for IQ comparison.

**TABLE 1:** Scan parameters used in brain and body protocols for this study in CT_p_/CT_s_ scanners.

	Brain	Body
kVp	120	120
Pitch	0.3	0.8
Slice thickness (mm)	2	2
Reconstructed FOV (mm)	350	500
Convolution kernel (CT_p_/CT_s_)	Standard/Hr38	Standard/Br38
CTD_Ivol_ (mGy)	60, 70, 80/×(scale factor)	5, 10, 15, 20, 30, 40/×(scale factor)
Reconstruction methods CT_p_ CT_s_	FBP;iDose4(1–5);IMR(1–3) FBP;SAFire(1–5)	FBP;iDose4(1–6);IMR(1–3) FBP;SAFire(1–5)

### 2.2 Rescaling NPS at different protocol between scanners using FBP reconstruction

Water phantoms were scanned at the reference CTDI_vol_ values of 32 cm water equivalent diameter in the body protocol and 16 cm water equivalent diameter in the brain protocol for the CT_p_ scanner. NPS for the CT_p_ scanner was measured (as described in Equation [ 1] in 2.3.1) and used to adjust the CTDI_vol_ value at CT_s_ scanner, ensuring that the area under the curve (AUC) of the NPS (AUC_NPS_) of CT_s_ matched the AUC_NPS_ of CT_p_. The mean of the resulting CTDI_vol_(CT_s_)/CTDI_vol_(CT_p_) ratio was used as a scale value, derived from multiple scans using the brain, head and neck, lung, abdomen, and pelvis protocols, to re‐scale the scanned CTDI_vol_ (5, 10, 15, 20, 30, 40 mGy in body protocol and 60, 70, 80 mGy in brain protocol) from CT_p_ to CT_s_ for IQ measurements.

### 2.3 IQ metrics

**FIGURE 1:**
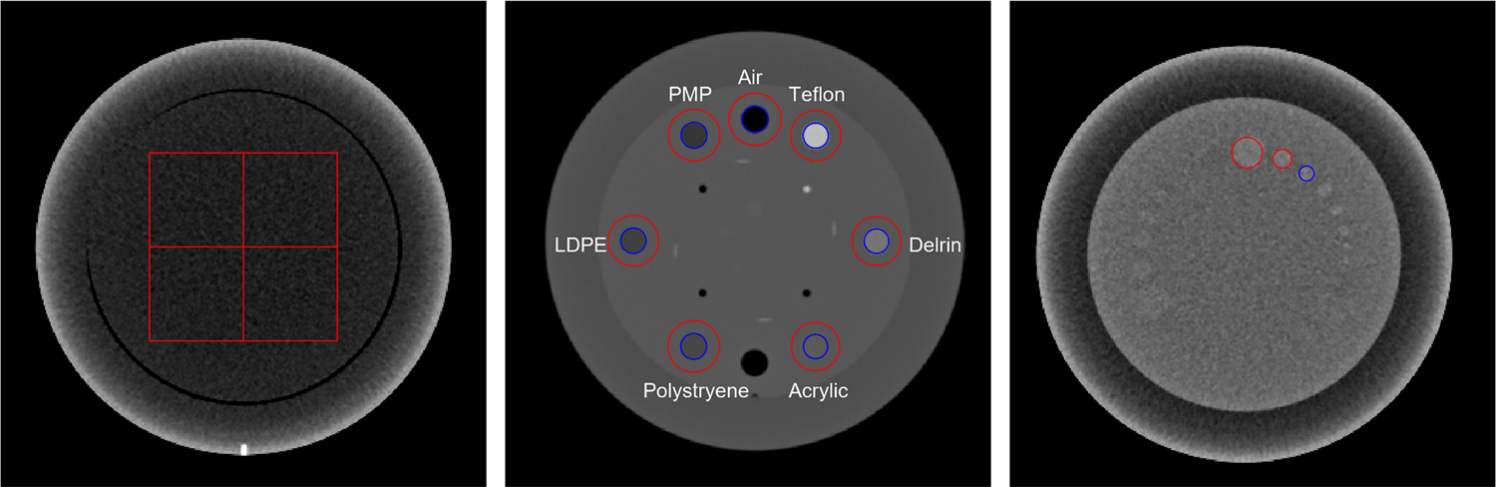
(a, left) CTP486 uniformity module with the overlaid ROIs; (b, middle) CTP404 module with seven sensitometry sample inserts; (c, right) CTP515 low contrast module, the 3rd diameter (blue) of the 1.0% supra‐slice was used in low contrast detectability (Section 2.3.4).

Figure 1 illustrates the CTP486, CTP404, and CTP515 modules of the Catphan phantom and the region of interest (ROI) and material inserts used for computing the IQ metrics

#### 2.3.1 Noise and texture measured from NPS

The 2D NPS(*f_x_*, *f_y_*) was calculated by taking the square of the 2D Fourier transform of the ROI in the uniformed section of the Catphan phantom (CTP486) or the 20 and 30 cm water phantoms for NPS_p_ normalization in 2.2 above. The formula used is as follow^6^:

(1) $${\mathrm{NPS}}_{2D}\left(f_{x},f_{y}\right)\;=\frac{\Delta x\Delta y}{N_{x}N_{y}}\;\sum_{i\;=\;1}^{N_{\mathrm{ROI}}}{\left|{\mathrm{FFT}}_{2D}\left[I_{{\mathrm{ROI}}_{i}\;}-P_{{\mathrm{ROI}}_{i}}\right]\right|}^{2}$$

where *f_x_* and *f_y_* are spatial frequency in the *x* and *y* direction, respectively; ∆*x* and ∆*y* are the pixel sizes in *x* and *y* direction, respectively; *N_x_* and *N_y_* are the number of pixels in *x* and *y* direction of the ROI; *N*_ROI_ is the number of ROIs; *I*_ROIi_ is the mean pixel value in ROI*_i_*; *P*_ROI_ *_i_* is the 2nd order polynomial fit of *I*_ROI_ *_i_*.

The noise, measured as the standard deviation (SD) of the ROI in Hounsfield units (HU), and the magnitude of the NPS peak (NPS_p_, peak intensity in HU^2^∙mm^2^) were recorded for noise comparison. The spatial frequency of the NPS peak (FREQ_p_ in mm^−1^) and the weighted average spatial frequency of NPS curve (FREQ_m_ in mm^−1^) were recorded for texture comparison.

#### 2.3.2 Contrast and CNR

The scanned images of the CTP404 module of the Catphan phantom were analyzed to calculate the contrast and CNR for the seven sensitometry samples (Polystyrene, Acrylic, LDPE, Derlin, PMP, Air, and Teflon). Contrast was measured as the difference in HU between the insert and the surrounding background material. The CNR was calculated by dividing the contrast by the STD of the background noise. This study presented the overall effective CNR of all visible rods within the insert, calculated using the average composite image of all analyzed slices of the CTP404 module.

#### 2.3.3 Task‐based detectability index

Two low object‐to‐background contrast inserts were evaluated: one was the acrylic insert with a diameter of 12 mm and a contrast difference, ∆HU (S_obj_–S_back_) of 25 HU in the CTP404 module, and the other one was a 7 mm diameter insert (nominally 8 mm according to the phantom reference manual, but measurable as 7 mm in the axial CT image) with ∆HU of 10 HU in the 1.0% super‐slice of the CTP515 low contrast module of the Catphan phantom. The detectability of the two inserts were denoted as d′(12 mm;25HU) and d′(7 mm;10HU) throughout the study.

Spatial resolution was quantified by computing the TTF, a quasi‐linear analog to the MTF, as described in detail by Richards et al.^16^ Richard's analysis involved examining the edge of the disk objects to establish the edge spread function (ESF), which was then differentiated to obtain the line spread function (LSF). The TTF of the objects was determined from the modulation transform of the LSF. The calculation of the LSF from the ESF for TTF computation utilized the techniques introduced by Ott et al.^17^ The approach by Ott involved incorporating two Gaussian functions into a sigmoid function to account for “overshoots” resulting from edge enhanced filters. This method was employed in the current study to assess low‐contrast materials with CNR < 15.

The non‐prewhitening observer model with eye filter (NPWE),^18^ representing the response of the human eye, was calculated using the following equation:

(2) $$\begin{array}{ccc} & & {\left(d_{NPWE}^{\prime }\right)}^{2}\\ & & \;=\frac{{\left[\int \;\;\;\int TTF^{2}\left(\mu ,\upsilon \right)W_{Task}^{2}\left(\mu ,\upsilon \right)E^{2}\left(\mu ,\upsilon \right)d\mu d\upsilon \right]}^{2}}{\int \;\;\;\int NPS\left(\mu ,\upsilon \right)TTF^{2}\left(\mu ,\upsilon \right)W_{Task}^{2}\left(\mu ,\upsilon \right)E^{4}\left(\mu ,\upsilon \right)d\mu d\upsilon } \end{array}$$

The task function $W_{Task}^{2}\left(\mu ,\upsilon \right)$ is defined by the Fourier transform of the difference of the hypotheses of object present (*h*_1_) and object absent (*h*_2_):

(3) $$W_{Task}^{2}\;\left(\mu ,\upsilon \right)=\left|FFT\left[h_{1}\left(x,y\right)-h_{2}\left(x,y\right)\right]\right|$$

The eye filter *E*(μ,υ) was the visualization function defined by Eckstein.^18^

#### 2.3.4 Performance of low contrast detectability using IR methods

To evaluate the overall effectiveness of low contrast detectability and compare two different hybrid statistical IR methods and one model‐based IR method, the d' value of the smaller diameter and ∆HU d'(7 mm;10HU) were further correlated with the AUC of the receiver operating characteristic curve (ROC) by assuming a symmetrical binomial distribution.^19^

(4) $$AUC\;=\frac{1}{2\left[1+\mathrm{erf}\left(\frac{d^{\prime }}{2}\right)\right]}\;$$

### 2.4 Analysis

An iQmetrix‐CT software developed by Greffier et al.^20^ was used to compute NPS, Contrast, CNR, NPWE detectability index of the CT images acquired by the scanners from two manufactures. A Spearman correlation (using XLSTAT software) was applied in evaluating the best fit of scanned reconstruction method from iDose‐3 (the clinical reference reconstruction) at CT_p_ to the SAFIRE level at CT_s_. Higher correlation values across all the IQ metrics evaluated were considered indicative of a better fit of the SAFIRE level in CT_s_ to iDose‐3 in CT_p_.

## 3 RESULTS

### 3.1 Normalization of NPS at different protocol using FBP reconstruction

**FIGURE 2:**
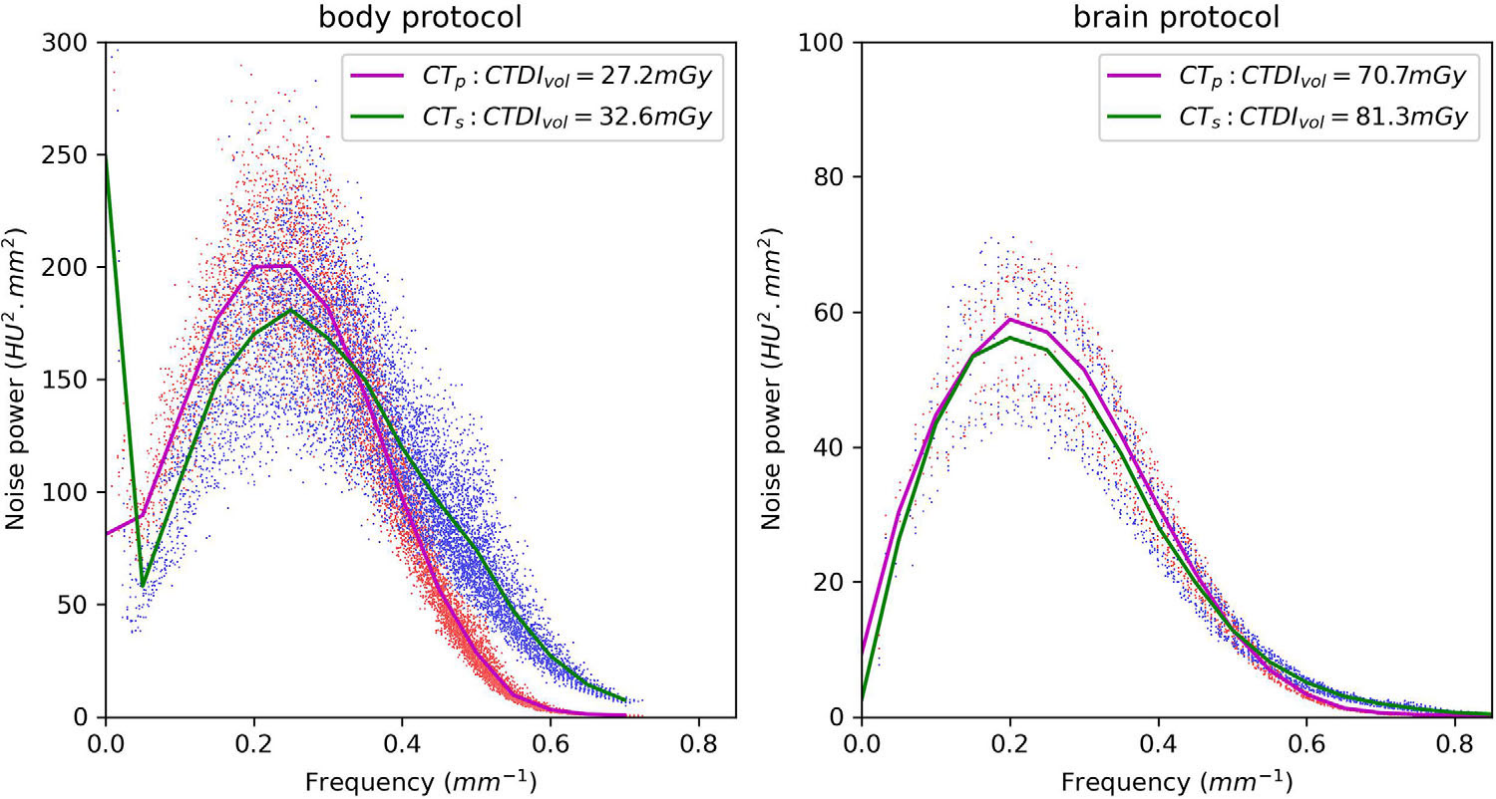
Matching of the AUC_NPS_ from two scanners by scaling the CTDI_vol_ at body scan of 30 cm water phantom (a, left) and brain scan of 20 cm water phantom (b, right), respectively.

In water phantoms (20 and 30 cm), across different protocols for head/brain (pitch = 0.3) and lung to pelvis (pitch = 0.8), with slice thickness ranging from 1 to 3 mm, and reference CTDI_vol_ values from 7 to 35 mGy for body site and 70 mGy for brain, comparison of the AUC_NPS_ indicated that scanned images from the CT_s_ scanner needed to increase CTDI_vol_ by 10%−25% compared to the scanned images from the CT_p_ scanner. Figure 2 demonstrates the comparisons of NPS from the scanned images using brain and pelvis protocols under FBP reconstruction. For all the IQ metrics studies, a scale value of 1.15 was used to rescale the CTDI_vol_ values from CT_p_ scanner for use in the scans at the CT_s_ scanner.

### 3.2 IQ metrics: Noise, texture, contract, CNR, detectability

**FIGURE 3:**
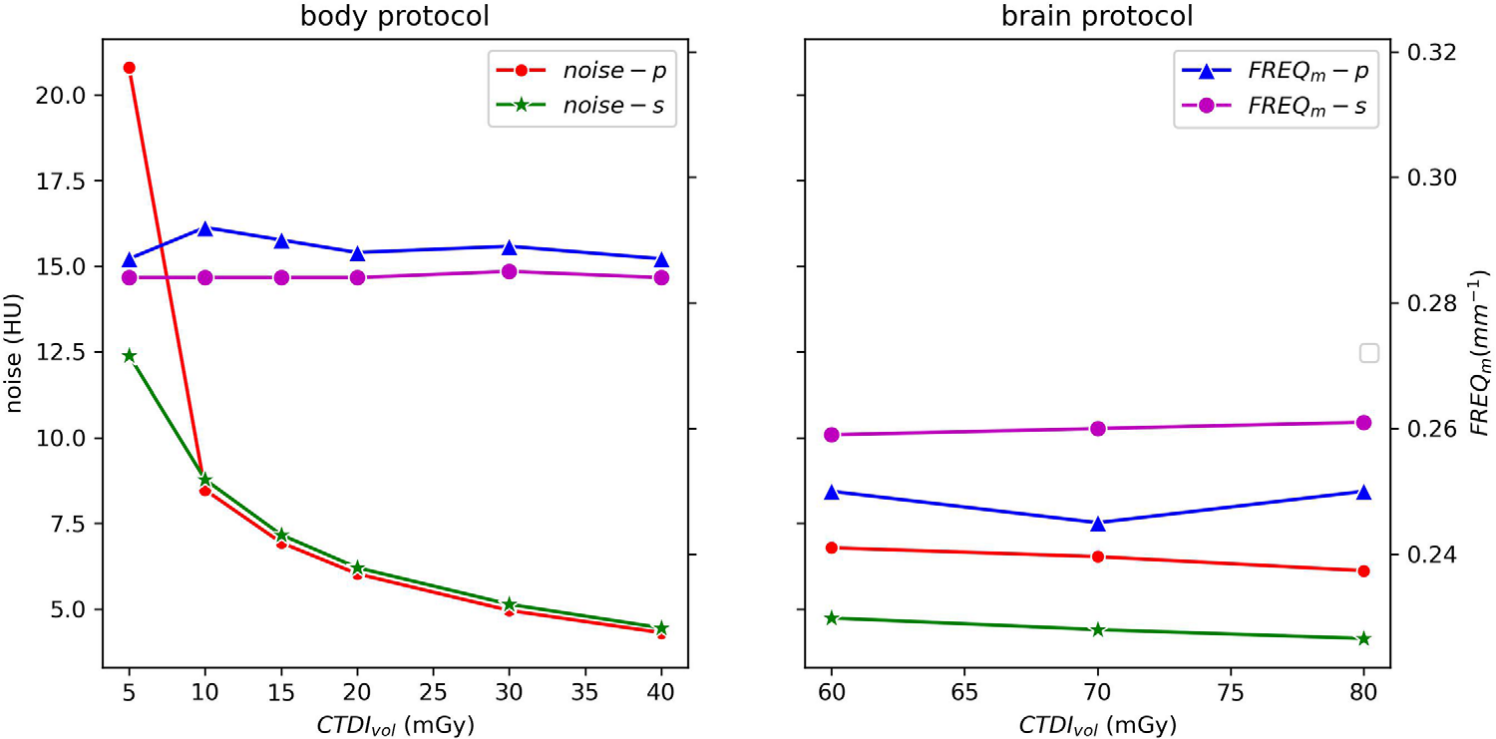
(a, left) The noise magnitude and (b right) average frequency (FREQ_m_) obtained from NPS analysis of uniform water phantom images from the scanners (CT_p_ and CT_s_) versus different CTDI_vol_ values. The same scanned parameters (listed in Table 1) and filter back projection (FBP) reconstruction were used for both scanners.

Figure 3 shows the noise magnitude and average frequency (FREQ_m_) derived from NPS analysis of uniform water phantom images scanned by the two scanners (CT_p_ vs. CT_s_) at different CTDI_vol_ for both brain and body, using a fixed reconstruction method of FBP. Except for the image from the low dose (5 mGy) of the CT_p_ scanner, the noise magnitude was roughly proportional to the inverse square root of CTDI_vol_. The texture level (FREQ_m_) had very little dependency on CTDI_vol_ under the same protocol.

Table 2. summarizes the contrast and CNR of the seven sensitometry sample inserts. The mean value and STD of contrast and CNR using different scanned CTDI_vol_ values and different reconstruction IR algorithms were calculated per scanner and per protocol. The STD of the contrast was small indicated that the contrast of the insert was independent of CTDI_vol_ and the IR algorithms. The STD of the CNR was larger compared to the STD of contrast due to noise varied with CTDI_vol_ and IR.

**TABLE 2:** Mean ± SD (standard deviation) of contrast (CNT) and CNR over all the CTDI_vol_ and IR combination per scan anatomical site from two scanners for the seven insert materials within the test phantom.

	Polystyrene	Acrylic	LDPE	Derlin	PMP	Air	Teflon
CNT(body, CT_p_)	−128 ± 1.3	25 ± 0.9	−183 ± 1.3	245 ± 1.1	−270 ± 1.5	−1055 ± 1	814 ± 2.3
CNT(body, CT_s_)	−135 ± 0.5	26 ± 0.4	−195 ± 0.6	263 ± 0.6	−288 ± 0.6	−1122 ± 1	870 ± 1.2
CNT(brain, CT_p_)	−131 ± 0.3	25 ± 0.3	−189 ± 0.4	193 ± 0.3	−278 ± 0.2	−1085 ± 1	763 ± 0.4
CNT(brain, CT_s_)	−156 ± 0.7	29 ± 0.6	−223 ± 0.6	298 ± 1.8	−329 ± 0.6	−1146 ± 1	986 ± 4.6
CNR(body, CT_p_)	71.4 ± 27	14 ± 5.7	97 ± 35.4	103 ± 28.9	134 ± 48.4	284 ± 73.5	197 ± 45.5
CNR(body, CT_s_)	50 ± 12.2	13 ± 5.2	60 ± 11.1	79 ± 14.1	78 ± 12.9	107 ± 3.1	98 ± 5.2
CNR(brain, CT_p_)	93 ± 12.2	19 ± 2.7	123 ± 13.3	59 ± 5.9	189 ± 25.4	403 ± 23.7	164 ± 19
CNR(brain, CT_s_)	60 ± 3.0	11 ± 0.8	71 ± 1.4	92 ± 3.6	103 ± 3.8	158 ± 6.0	133 ± 1.4

**FIGURE 4:**
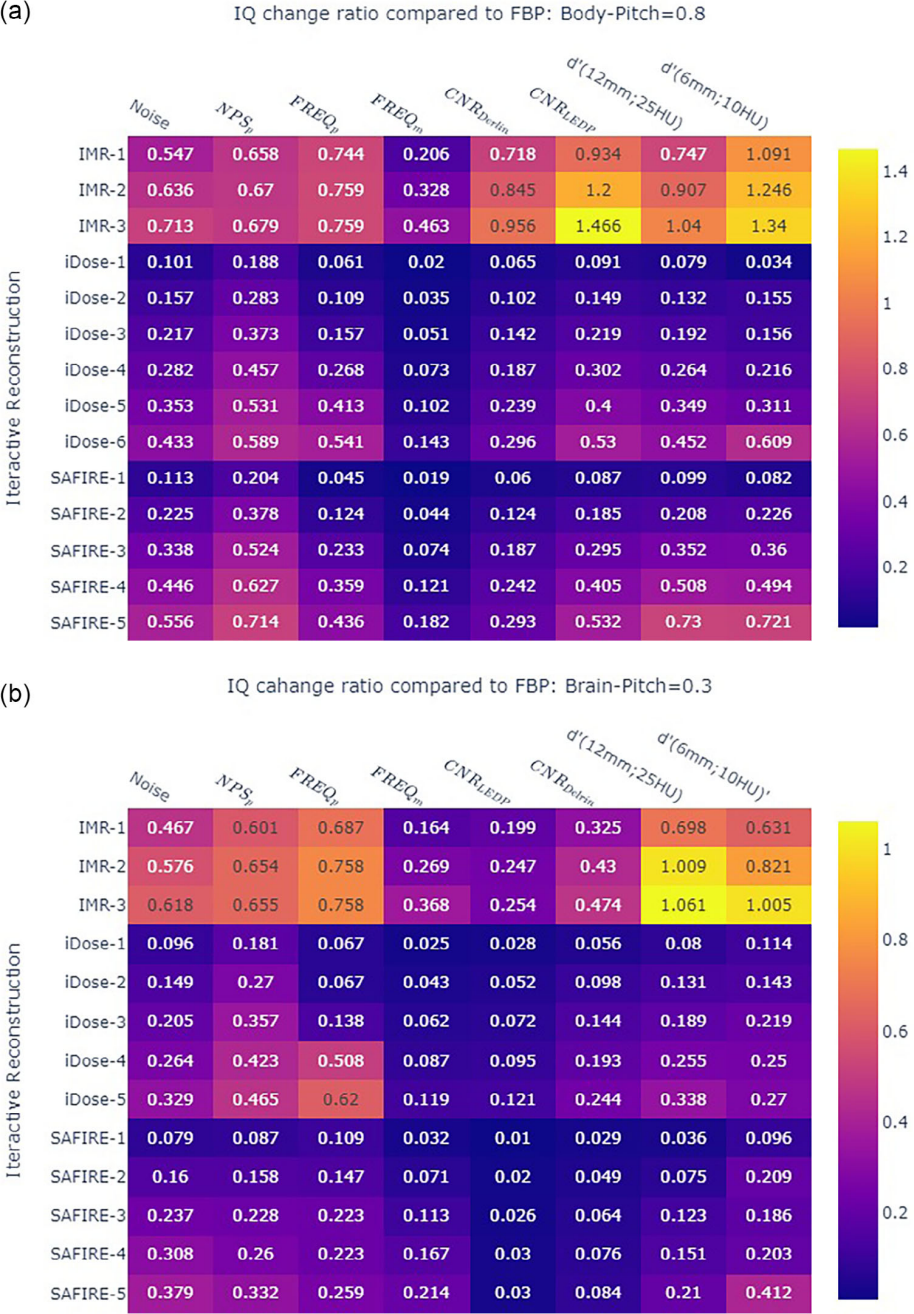
2D matrix displays of the ratio of the IQ metrics, showing the changes by IRs compared to FBP, with a color bar to code the heatmap of the ratio value. (a) Shows the comparison for the body protocol, and (b) shows the comparison for the brain protocol. FBP, filter back projection; IQ, image quality; IR, iterative reconstruction.

IR has non‐linear impact on all the IQ metrics. Using the IQs obtained from FBP reconstruction as reference, the ratio of the IQ metrics difference between the IR reconstruction and the FBP reconstruction ((IQ_IR_−IQ_FBP_)/IQ_FBP_) was analyzed. Figure 4 shows the heatmap distribution with annotated value equal to the average IQ change ratio from all the CTDI_vol_ settings. Only the CNR of LEDP and Derlin sample inserts are shown in the heatmap figure to save the size. The results showed that the degree of the change ratio was moderate (<0.6) at both iDose and SAFIRE reconstruction methods. The change ratio was stronger (>0.6, except FREQ_m_) when images were reconstructed by the IMR. When iDose‐3 was considered the reference of the protocols at CT_p_ scanner, SAFIRE‐2 had a closer match at the CT_p_ scanner for the body protocol at CTDI_vol_ range of 5−40 mGy (Spearman correlation of iDose‐3 vs. SAFIRE 1−5: 0.985, 0.989, 9.986, 0.979, 0.969; *p*‐values were all less than 0.0001). There was no significant difference in brain protocol in matching iDose‐3 with SAFIRE‐1–4 at CTDI_vol_ range of 60−80 mGy (Spearman correlation of iDose‐3 vs. SAFIRE 1−5: 0.935, 0.934, 0.935, 0.935, 0.933; *p*‐values were all less than 0.0001). Table 3 summarizes the results of the IQ metrics after matching.

**TABLE 3:** The mean IQ metric values at the CTID_vol_ range in this study for iDose‐3 of body (denoted *) and brain (denoted ^) protocols at CT_p_ scanner and the SAFire‐2 and SAFire‐3 of body and brain protocols at CT_s_, respectively.

	Noise	NPS_p_	FERQ_p_	FERQ_m_	CNR_LEDP_	CNR_Derlin_	d′(12 mm;25HU)	d′(6 mm;10HU)
iDose‐3*	6.72	113.3	0.20	0.27	57.7	86.7	9.21	3.21
SAFire‐2*	5.70	80.1	0.21	0.27	47.5	76.8	10.41	4.14
iDose‐3^	5.15	54	0.19	0.23	56.8	87.0	7.88	3.27
SAFire‐3^	3.38	26	0.16	0.23	60.7	92.8	14.85	5.19

### 3.3 Performance of low contrast detectability using IR methods

**FIGURE 5:**
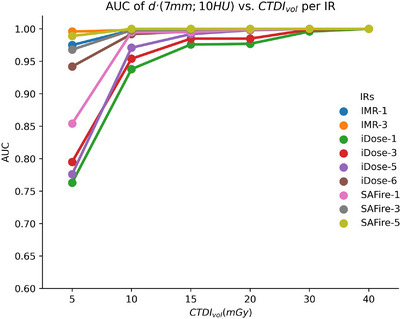
The AUC observer performance plot for the detectability of the lower contrast material. SAFIRE was better at low CTDI_vol_ than iDose. Model‐based iterative reconstruction (IMR) was the best.

After converting the lower d′ (diameter = 7 mm and ∆HU = 10) of this study to AUC, Figure 5 was created to demonstrate the low contrast detectability using different IR methods. All IRs had AUC of d′(7 mm;10HU) over 0.95 at CTDI_vol_ of 15 mGy or higher. The AUC of d′(7 mm;10HU) using IMR, SAFIRE, and iDose level > 3 was higher than 0.95 when CTDI_vol_ was down to 10 mGy. When CTDI_vol_ was as low as 5 mGy, the AUC of d′(7 mm;10HU) using iDose method (level 1−6) and the weakest SAFIRE (level 1) were smaller than 0.95. The lowest CTDI_vol_ (60 mGy) for brain protocol at this study was sufficient to detect the lower contrast of 6 mm/10 ∆HU such that all IRs had AUC higher than 0.95.

## 4 DISCUSSION

This study used quantitative IQ metrics recommended by TG 233 to compare the scanners from two manufacturers and develop a technique to standardize clinical CT simulation scan protocols. While the fundamental principles of CT image formation, including data acquisition, processing, and reconstruction algorithms, are consistent across CT systems, different manufacturers implement proprietary technologies that can lead to variability in the final IQ. Despite using similar acquisition techniques and reconstruction methods, the noise texture, spatial resolution, and overall image characteristics can differ across CT scanners from different vendors. These differences stem from the advanced automation and proprietary implementations of various aspects, such as data calibration, beam‐hardening correction, and image processing algorithms. As a result, there is no simple or straightforward way to match IQ across scanners, necessitating more nuanced methods to standardize and translate clinical CT simulation scan protocols between different CT systems. Since this study aimed to translate clinical scanned protocols between scanners from different manufacturers for RT simulation and treatment purposes, the tube potential (kV) was fixed at 120 kV. Additionally, the pitch, collimator, reconstruction kernel, and slice thickness were fixed separately for brain and body categories. This separation accounted for the differences in bowtie filter design and the reference CTDIvol definitions, which are specific to head (16 cm diameter) and body (32 cm diameter) imaging.

As the study was conducted using phantoms with fixed diameters, ATC was not utilized. Instead, a range of CTDI_vol_ values from low to high, along with different IR strengths, were scanned and reconstructed for IQ analysis. Among the IQ metrics in this study, the NPS quantifies the noise present at various spatial frequency, characterizing the noise texture of an image. It can be considered as a signature of an image produced from a specific scanner at the specific scanned parameters. Given the potential variance in actual CTDI_vol_ delivered by different scanners, normalized NPS offers a more reliable benchmark for comparing scanner discrepancies resulting from different non‐linear reconstruction kernels or IR. Comparisons across using different clinical protocol (brain, Head and neck, lung, abdomen, pelvis) with different reference mAs (corresponding to different CTDI_vol_), pitch, and slice thickness, CT_s_ scanner needed 5%−20% higher CTDI_vol_ to display the same level of noise compared to CT_p_ scanner. Considering the typical 20% tolerance in delivered CTDI_vol_, these differences were deemed acceptable.

In evaluating the IQ metrics at different clinical CTDI_vol_ levels and applying various IR algorithms, the IR method that reconstructed images with lower noise, reduced texture alteration as compared to FBP reconstructed images, with higher CNR, and low contrast detectability was considered superior. The IMR method demonstrated excellent noise reduction, highest CNR, and improved low contrast detectability. However, there were shifts in FREQ_p_ from the standard (FBP) value of 0.25 mm^−1^ to 0.05–0.07 mm^−1^ and FREQ_m_ from the standard (FBP) value of 0.29 mm^−1^ to less than 0.2 mm^−1^. The overall NPS shifted toward the lower frequency side may result a patchy or blotchy appearance. There was clinical study demonstrated the blotchy image appearance of the chest image after model‐based reconstruction.^21^ The utilization of IMR in clinical CT images requires the validation by clinicians.

The weak (level 1−2) to moderate strength (3–4) of SAFIRE was close to the same hybrid statistical‐based IR of iDose (level 1−6). Each level increase in SAFIRE was close to two level increase at the iDose. In the stronger strength of SAFIRE (level 4−5), the magnitude of the IQ metrics change from FBP was similar to the magnitude of the IQ metric change at IMR. The using of the strong SAFIRE needs to be cautious if blotchy image was not desired.

Interestingly, the contrast of various insert materials, ranging from low‐density acrylic to high‐density Teflon, remained consistent within the same protocol (body or brain) and was independent of CTDI_vol_ or IR strength. While the HU values were similar between CT_p_ and CT_s_ for the body protocol (differences < 50 HU, except for air), significant differences exceeding 100 HU were observed for high‐density materials like Teflon and Derlin when comparing body and brain protocols. These differences could be attributed to variations in bowtie filter design and system calibration between head and body imaging. The impact of such HU discrepancies on dose calculations in clinical radiation treatment should be further evaluated. Nevertheless, the low contrast small object detectability (d′ [6 mm;10HU] in this study) remained reasonably high, providing scanned CTDI_vol_ was not extremely low.

This study did not utilize ATC evaluation. Instead, a range of clinical CTDI_vol_ values were scanned at the 20 cm diameter phantom. The range used in the body protocol included the lower dose range (5–15 mGy) and higher dose rage (30–40 mGy) to the scanned size of 20 cm diameter. It appeared that using FBP reconstruction, the noises were roughly proportional to inverse square of CTDI_vol_ except the scanned image applied 5 mGy from CT_p_ (Figure 3 left). This discrepancy may be due to the low dose level being too small for the aging CTp scanner, which is scheduled for retirement in a year, resulting in electronic noise that could not be ignored. The scanned images from CT_s_, which installed most recently, did not show a dramatical noise increase at CTDI_vol_ as low as 5 mGy. Clinically, the adult brain protocols of both scanners use a fixed CTDI_vol_ of 70 mGy. The studied CTDI_vol_ range (60–80 mGy) was relatively small in comparing to the studies of body protocol, and the results showed smaller IQ metrics change of less than 0.3 at iDose and SAFIRE below or equal level 3.

The Capthan phantom and ACR phantom consist of various modules designed for measuring IQ or evaluate task performance. Their standard geometry facilitates the adaptation of IQ metrics and Task functions by software, allowing for comprehensive testing under different scanned and reconstructed condition. While these tests are ideal for quantifying a CT scanner's system performance during the pre‐clinical phase, they are not suitable for accessing ATC performance. Clinical CT images, on the other hand, exhibit more complex material compositions and asymmetrical geometry, leading to various degree of beam hardening, softening, photon starvation effects that can impact image reconstruction performance and CT IQ. Evaluating CT IQ during the clinical phase is crucial for standardizing CT scan protocols. To address this, an automatic assessment algorithm was developed to evaluate CT IQ in Rando phantom and patient CT images, with the results of the evaluation being published separately.^22^

Using the clinical protocols at CT_p_ as reference, appropriate scanned parameters of the clinical protocols at CT_s_ were identified based on the steps of (a) separated study into body and brain protocols, (b) rescale NPS between scanners, (c) varied CTDI_vol_ and IR strength, (d) evaluating the IQ change, and (e) matched the IR strength level. When assessing the comprehensive performance of a CT system and its operation, it is essential to include the evaluation of ATC. However, this study did not focus on evaluating the ATC of both scanners. Another study has been conducted to assess the accuracy of translating clinical phase CT protocols among different scanners and manufacturers.^23^, ^24^ Although the current study was performed on phantoms without ATC, it provided a preliminary guide for translating clinical protocols from one scanner to another.

## 5 CONCLUSION

This study utilized a list of IQ metrics to quantitatively analyze the difference of IQs at different anatomy protocol versus CTDI_vol_ and IR at two scanners. The mapping of the IQ change form FBP to IR allowed to evaluate the strength of IR at different levels. The closest match of reference protocol in the reference iDose‐3 level of simulator CT_p_ to simulator CT_s_ which uses a different IR of SAFIRE was identified. These matches were able to combine further with ATC to translate the master clinical protocols of CT_p_ scanner to another scanner (CT_s_) from different manufacturer.

## AUTHOR CONTRIBUTIONS

Each co‐author has participated sufficiently in the study of this submission and all authors have approved for this publication. Contribution from each author is detailed below. Hsiang‐Chi Kuo conceived of the presented idea, developed the theory, and performed the computations, wrote the manuscript with support from the co‐authors. Usman Mahmood conceived of the presented idea, contributed to the design and implementation of the research. Assen S. Kirov contributed to the design and implementation of the research. Trevin Trotman contributed to sample preparation and managed CT protocols. Shih‐Chi Lin performed the experiments, derived the models, and analyzed the data. James G. Mechalakos provided discussion to clinical impact of CT images quality. Cesar Della Biancia provided discussion to clinical impact of CT images quality. Laura I. Cerviño contributed to the final version of the manuscript and supervised the project. Seng Boh Lim investigated and supervised the findings of this work. All authors discussed the results and commented on the manuscript.

## CONFLICT OF INTEREST STATEMENT

The authors declare no conflicts of interest.
